# Women’s perspectives on the use of artificial intelligence (AI)-based technologies in mental healthcare

**DOI:** 10.1093/jamiaopen/ooad048

**Published:** 2023-07-08

**Authors:** Meghan Reading Turchioe, Sarah Harkins, Pooja Desai, Shiveen Kumar, Jessica Kim, Alison Hermann, Rochelle Joly, Yiye Zhang, Jyotishman Pathak, Natalie C Benda

**Affiliations:** Columbia University School of Nursing, New York, New York, USA; Columbia University School of Nursing, New York, New York, USA; Department of Biomedical Informatics, Columbia University, New York, New York, USA; Cornell University, Ithaca, New York, USA; Department of Population Health Sciences, Weill Cornell Medicine, New York, New York, USA; Department of Psychiatry, Weill Cornell Medicine, New York, New York, USA; Department of Obstetrics and Gynecology, Weill Cornell Medicine, New York, New York, USA; Department of Population Health Sciences, Weill Cornell Medicine, New York, New York, USA; Department of Population Health Sciences, Weill Cornell Medicine, New York, New York, USA; Department of Psychiatry, Weill Cornell Medicine, New York, New York, USA; Columbia University School of Nursing, New York, New York, USA

**Keywords:** artificial intelligence, mental health, women’s health, trust, bioethics

## Abstract

This study aimed to evaluate women’s attitudes towards artificial intelligence (AI)-based technologies used in mental health care. We conducted a cross-sectional, online survey of U.S. adults reporting female sex at birth focused on bioethical considerations for AI-based technologies in mental healthcare, stratifying by previous pregnancy. Survey respondents (*n* = 258) were open to AI-based technologies in mental healthcare but concerned about medical harm and inappropriate data sharing. They held clinicians, developers, healthcare systems, and the government responsible for harm. Most reported it was “very important” for them to understand AI output. More previously pregnant respondents reported being told AI played a small role in mental healthcare was “very important” versus those not previously pregnant (*P* = .03). We conclude that protections against harm, transparency around data use, preservation of the patient-clinician relationship, and patient comprehension of AI predictions may facilitate trust in AI-based technologies for mental healthcare among women.

## BACKGROUND AND SIGNIFICANCE

The growing availability of big data in healthcare, together with the increasing sophistication of artificial intelligence (AI) models, has created new opportunities to improve outcomes in all aspects of mental healthcare and services, including prevention, management, and treatment.^1^ In particular, there is great potential to apply AI to predict mental health disorders due to their widespread prevalence and inadequate diagnosis and treatment. For example, an estimated 21 million US adults experienced a major depressive disorder in 2020, but nearly 60% did not receive treatment.^2^

Despite a deluge of articles discussing the ethics of AI^3–5^ and strategies for presenting AI to clinicians,^6^^,^^7^ there has been little discussion of patient perspectives and the role of AI-based technologies in shared clinical decision-making.^7^ Ethical questions lie at the heart of many of the biggest challenges to implementation of AI in clinical practice when considering patients’ needs.^8^ Trust is 1 important aspect of these ethical considerations, and a fundamental requirement for effective medical care, with or without AI, to be delivered.^3^^,^^4^ A practical understanding of how to ethically conduct patient-facing AI research in mental health is critically needed.

There is a particularly unique need to understand ethical considerations for AI in mental health care among women. The prevalence of depression is twice as high in women compared to men.^9^ Moreover, ethical considerations become more complex when women’s mental health intersects with reproductive health (eg, perinatal mood and anxiety disorders, or PMADs).^10^^,^^11^ Pregnancy brings physiologic changes and levels of healthcare interaction that are distinct for otherwise healthy young adults. Anxieties regarding how reproductive data are used are particularly pronounced among pregnancy-capable persons subjected to new and changing reproductive rights in the post-Roe versus Wade Era.^11^ Therefore, now more than ever, it is important to assess whether experiences of previous pregnancies affect perceptions of AI in mental health care.

In this study, we build on prior studies examining attitudes toward AI in healthcare in general,^12^ and attitudes among pregnant people toward AI,^13^ by conducting a deeper examination of bioethical concerns on the use of AI in mental health care among individuals reporting female sex at birth.

## OBJECTIVE

We conducted a subgroup analysis of attitudes toward the use of AI-based technologies for mental healthcare among individuals reporting female sex at birth, stratifying by self-reported previous pregnancy status.

## MATERIALS AND METHODS

### Sample and study design

We conducted a cross-sectional survey of US adults in September 2022. We used the online survey recruitment platform, Prolific,^14^ to recruit survey respondents into the study. Survey respondents were verified Prolific users aged 18 or older who were fluent in written and spoken English. The sample was balanced on age, gender, and race to reflect US demographic distributions.^15^ We recruited 530 survey respondents, of whom 30 did not begin the survey after reading the informed consent document, resulting in a total of 500 respondents. All 500 respondents finished the survey (zero incomplete responses) over a median time of 15 minutes and 24 seconds. For this analysis, we only examined responses of a subset of survey respondents reporting female sex at birth to focus on pregnancy-capable persons. The Weill Cornell Medicine (WCM) Institutional Review Board approved this study.

### Survey items

Survey questions (Supplementary File S1) were derived from prior work on examining AI in general health contexts but not mental health^12^ and focused on: (1) general perspectives on the use of AI for mental healthcare (including knowledge, data sharing, transparency, explainability, responsibility, and the effect of AI on trust in mental health professionals), (2) comfort with the use of AI in place of mental health professionals for various tasks, and (3) concerns regarding the use of AI for mental healthcare. Additionally, a fourth set of questions evaluated the relative importance of 6 bio-ethical constructs to survey respondents. Constructs were drawn from prior research in AI ethics broadly,^5^ ethics on the use of consumer-generated data,^16^ ethics on the use of AI in psychiatry,^17^ and maternal mental health ethics.^10^^,^^18^

Survey items were iterated upon through consultation with experts in machine learning, human-centered design, and psychiatry. Definitions of AI, mental health, clinical depression, and bipolar disorder using lay terms were provided at the beginning and throughout the survey. Throughout the survey, 2 “attention check” questions were included to ensure survey respondents were fully reading each question and thoughtfully responding.^15^

Finally, survey respondents completed survey questions regarding sociodemographic characteristics, health literacy,^19^ subjective numeracy,^20^ preferences for involvement in medical decision-making,^21^ mental health history, and self-reported previous pregnancy status.

### Data collection

We created the survey using a secure institutional instance of Qualtrics provisioned by WCM. Prolific invited eligible persons to participate, and interested parties followed a link to the Qualtrics survey. Survey respondents provided informed consent by reading an information sheet and clicking a box to indicate consent. Survey respondents were permitted to stop the survey at any time, and were permitted to navigate backwards and forwards while taking the survey. Survey respondents completing the survey were compensated at an hourly rate of $13.60 consistent with Prolific policies.

### Statistical analysis

We computed basic descriptive statistics of all survey items to characterize the subgroup of the sample who reported female sex at birth. We used Wilcoxon rank-sum tests for continuous variables and Fisher’s Exact tests as appropriate to compare differences in sociodemographic characteristics and in all survey responses between survey respondents self-reporting a previous pregnancy versus not previously pregnant. Fisher's exact test for count data with simulated *P*-value (based on 2000 replicates) was used for larger contingency tables. The alpha value was set at 0.05. R version 4.2.1 (R Foundation for Statistical Computing, Vienna, Austria, 2022) was used for all analyses.

## RESULTS

Below we present selected results from this survey; detailed tables (including differences in survey responses by self-reported previous pregnancy status) are provided in Supplementary File S2.

### Sample characteristics

Two hundred fifty-eight survey respondents reported their sex assigned at birth as female and were included in this subgroup analysis (Table 1). Three survey respondents identified their current gender as male (1%). Compared to survey respondents not previously pregnant, those with previous pregnancies were significantly older (median age 56 vs 35) and a higher proportion rated their mental health as “excellent” (12% vs 5%) or “very good” (31% vs 19%).

**Table 1.** Characteristics of survey respondents reporting female sex at birth; overall and stratified by self-reported previous pregnancy status

	Overall (*n* = 258)	Previously pregnant (*n* = 140)	Not previously pregnant (*n* = 118)	*P*-value*
Age (median, IQR)	48 (32, 60)	56 (43, 61)	35 (24, 51)	**<.001**
Race				.62
Asian	12 (4%)	6 (4%)	6 (5%)	
Black or African American	35 (14%)	20 (14%)	15 (13%)	
White	200 (78%)	106 (76%)	94 (80%)	
Other/prefer not to answer**	11 (4%)	8 (6%)	3 (2%)	
Ethnicity				.62
Hispanic/Latino	17 (7%)	8 (6%)	9 (8%)	
Not Hispanic/Latino	241 (93%)	132 (94%)	109 (92%)	
Education				.57
Less than bachelor's degree	121 (47%)	69 (49%)	52 (44%)	
Bachelor's degree	93 (36%)	50 (36%)	43 (36%)	
More than bachelor's degree	44 (17%)	21 (15%)	23 (19%)	
Health literacy				.31
Adequate	196 (76%)	110 (79%)	86 (73%)	
Inadequate	62 (24%)	30 (21%)	32 (27%)	
Subjective numeracy				.70
Low subjective numeracy	162 (63%)	86 (62%)	76 (64%)	
High subjective numeracy	95 (37%)	53 (38%)	42 (36%)	
(Missing)	1	1	0	
Control preferences scale				.92
Make the final selection about which treatment I will receive	24 (9%)	14 (10%)	10 (8%)	
Make the final selection after seriously considering my doctor's opinion	114 (44%)	62 (44%)	52 (44%)	
Have my doctor and I share responsibility for deciding what treatment is best	101 (39%)	55 (39%)	46 (40%)	
Have my doctor make the final decision but consider my opinion	19 (8%)	9 (7%)	10 (8%)	
Leave all decisions regarding treatment to my doctor	0 (0%)	0 (0%)	0 (0%)	
Ever been told have mental illness				.05***
Yes	143 (55%)	68 (49%)	75 (64%)	
No	110 (43%)	69 (49%)	41 (35%)	
Prefer not to answer	5 (2%)	3 (2%)	2 (1%)	
Overall mental health rating				**.02**
Excellent	23 (9%)	17 (12%)	6 (5%)	
Very good	67 (26%)	44 (31%)	23 (19%)	
Good	84 (33%)	37 (26%)	47 (40%)	
Fair	62 (24%)	34 (24%)	28 (24%)	
Poor	20 (7%)	7 (5%)	13 (11%)	
Do not know	2 (1%)	1 (2%)	1 (1%)	

* Wilcoxon rank-sum test; Fisher's exact test for count data with simulated *P*-value (based on 2000 replicates); Fisher's exact test for count data. Bold indicates statistical significance at *P* < .05.

** Other racial groups included American Indian or Alaskan Native and Native Hawaiian or other Pacific Islander.

*** This *P*-value is .052 and is therefore above the threshold for statistical significance and not bolded.

### Perspectives on the use of AI in mental health care

Most survey respondents knew “a little bit” or “almost nothing” about the use of AI-based technologies in mental health care, but nearly half anticipated it would lead to improvements (Table 2). Most were comfortable sharing sensitive information with mental health providers, but only half were comfortable sharing it with AI chatbots or to train and test AI models. Nearly all thought it was somewhat or very important to know when AI played even a small role in diagnosis or treatment, and half were uncomfortable with AI that was not explainable even when it was highly accurate (98%). The majority felt mental health providers were responsible for medical errors resulting from AI, but most endorsed developers, health systems, and government agencies that make, implement, and approve the AI as the parties responsible for ensuring its safety.

**Table 2.** Attitudes toward the use of artificial intelligence (AI)-based technologies in mental healthcare among survey respondents reporting female sex at birth, overall and by self-reported previous pregnancy status; *n* (%)

	Overall (*n* = 258)	Previously pregnant (*n* = 140)	Not previously pregnant (*n* = 118)	*P*-value*
Knowledge of AI and how it could change mental healthcare				**.02**
Quite a lot	1 (1%)	1 (1%)	0 (0%)	
A fair amount	27 (10%)	18 (13%)	9 (7%)	
A little bit	137 (53%)	63 (45%)	74 (63%)	
Almost nothing	93 (36%)	58 (41%)	35 (30%)	
Effect of AI on mental healthcare in next 5 years				.40
Much better	11 (5%)	9 (7%)	2 (2%)	
Somewhat better	104 (40%)	54 (39%)	50 (42%)	
Minimal change	89 (34%)	45 (32%)	44 (37%)	
Somewhat worse	21 (8%)	13 (9%)	8 (7%)	
Much worse	4 (2%)	3 (2%)	1 (1%)	
Don't know	29 (11%)	16 (11%)	13 (11%)	
Comfort sharing sensitive information with a human mental health professional				.58
Very comfortable	101 (39%)	57 (41%)	44 (37%)	
Somewhat comfortable	105 (41%)	56 (40%)	49 (42%)	
Somewhat uncomfortable	42 (16%)	20 (14%)	22 (19%)	
Very uncomfortable	10 (4%)	7 (5%)	3 (2%)	
Don't know	0 (0%)	0 (0%)	0 (0%)	
Comfort sharing sensitive information with an AI chatbot				.96
Very comfortable	41 (16%)	23 (16%)	18 (15%)	
Somewhat comfortable	83 (32%)	44 (31%)	39 (33%)	
Somewhat uncomfortable	66 (26%)	34 (24%)	32 (27%)	
Very uncomfortable	64 (25%)	37 (26%)	27 (23%)	
Don't know	4 (1%)	2 (3%)	2 (2%)	
Comfort sharing sensitive information to help improve AI programs that treat disease				.63
Very comfortable	56 (22%)	28 (20%)	28 (24%)	
Somewhat comfortable	103 (40%)	60 (43%)	43 (36%)	
Somewhat uncomfortable	52 (20%)	26 (19%)	26 (22%)	
Very uncomfortable	32 (12%)	16 (11%)	16 (14%)	
Do not know	15 (6%)	10 (7%)	5 (4%)	
Importance of being told when AI has played a small role in mental health diagnosis or treatment				**.03**
Very important	134 (52%)	83 (59%)	51 (43%)	
Somewhat important	102 (40%)	50 (36%)	52 (44%)	
Not important	15 (6%)	5 (4%)	10 (9%)	
Do not know	7 (2%)	2 (1%)	5 (4%)	
Comfort with AI that is correct 90% of the time but not unexplainable				.90
Very comfortable	7 (2%)	4 (2%)	3 (3%)	
Somewhat comfortable	46 (18%)	26 (19%)	20 (17%)	
Somewhat uncomfortable	95 (37%)	54 (39%)	41 (35%)	
Very uncomfortable	105 (41%)	53 (38%)	52 (44%)	
Do not know	5 (2%)	3 (2%)	2 (1%)	
Comfort with AI that is correct 98% of the time but not unexplainable				.87
Very comfortable	29 (11%)	16 (11%)	13 (11%)	
Somewhat comfortable	70 (27%)	35 (25%)	35 (30%)	
Somewhat uncomfortable	82 (32%)	47 (34%)	35 (30%)	
Very uncomfortable	70 (27%)	39 (28%)	31 (26%)	
Don't know	7 (3%)	3 (2%)	4 (3%)	
Responsible parties for medical errors from AI				
Mental health professional	215 (83%)	121 (86%)	94 (80%)	.18
Company that made the computer program	87 (34%)	46 (33%)	41 (35%)	.79
Hospital or clinic that bought the computer program	73 (28%)	37 (26%)	36 (31%)	.49
Government agency that approved the computer program	55 (21%)	28 (20%)	27 (23%)	.65
Someone else	11 (4%)	4 (3%)	7 (6%)	.35
No one	1 (1%)	0 (0%)	1 (1%)	.46
Do not know	17 (7%)	7 (5%)	10 (9%)	.32
Responsible parties for checking if AI is safe				
Mental health professional	61 (24%)	36 (26%)	25 (21%)	.46
Company that made the computer program	171 (66%)	92 (66%)	79 (67%)	.90
Hospital or clinic that bought the computer program	149 (58%)	78 (56%)	71 (60%)	.53
Government agency that approved the computer Program	122 (47%)	57 (41%)	65 (55%)	**.02**
Someone else	4 (2%)	2 (1%)	2 (2%)	>.99
No one	9 (4%)	6 (4%)	3 (3%)	.51
Do not know	6 (2%)	3 (2%)	3 (3%)	>.99

* Fisher's exact test for count data with simulated *P*-value (based on 2000 replicates); Fisher's exact test for count data. Bolded values indicate significance at *P* < .05.

Compared to survey respondents with no previous pregnancies, survey respondents with a previous pregnancy: (1) more frequently knew “a fair amount” or “almost nothing” about AI in mental health care, (2) more often perceived it was “very” important to be told when AI has played a small role in mental health diagnosis or treatment, and (3) were less likely to agree the government was responsible for ensuring AI safety. There were no other significant differences in responses by self-reported previous pregnancy status.

### Comfort with AI in certain mental healthcare scenarios

**Figure 1.**
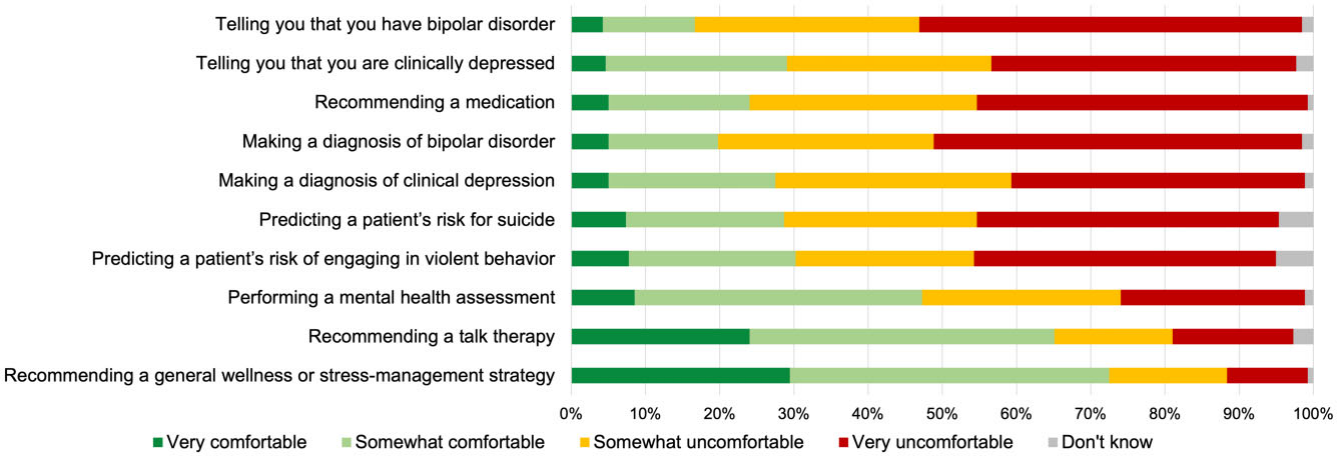
Survey respondents’ comfort with AI, instead of a mental health professional, performing mental health tasks (*n* = 258). Alt text: Horizontal stacked bar chart summarizing survey respondents’ level of comfortability with AI performing various tasks showing people are more comfortable with less serious tasks but less comfortable with more serious tasks.

Examining survey respondents’ comfort with AI performing various mental healthcare related tasks compared to a mental health professional, survey respondents were generally comfortable with AI recommending low-risk interventions such as general wellness strategies or talk therapy (Figure 1). However, the majority of survey respondents were somewhat or very uncomfortable with AI diagnosing disease or recommending medications. There were no significant differences in attitudes by self-reported previous pregnancy status.

### Concerns about AI in mental healthcare

**Figure 2.**
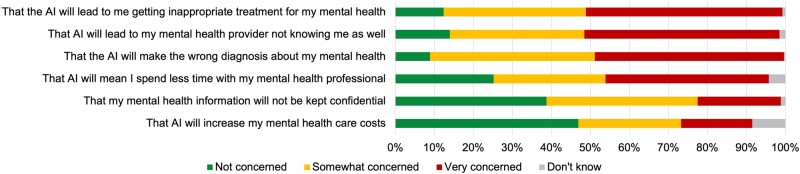
Level of concern about aspects of AI being used in mental health care (*n* = 258). Alt text: horizontal stacked bar chart summarizing survey respondents’ level of concerns about common pitfalls of AI showing survey respondents are less concerned about getting inappropriate treatment/diagnoses and more concerned about confidentiality and costs.

Half of the sample was “very” concerned that AI will lead to inappropriate treatment and to relationships with mental health providers being negatively impacted (Figure 2). Nearly half were concerned about AI making the wrong diagnosis. Fewer survey respondents were concerned about mental health care costs or confidentiality. There was a significant difference in responses to the concern that AI will mean spending less time with one’s mental health professional, with more survey respondents with no previous pregnanancies being “very” concerned (46%) versus those previously pregnant (39%; *P* = .032).

### Bioethical construct importance

**Figure 3.**
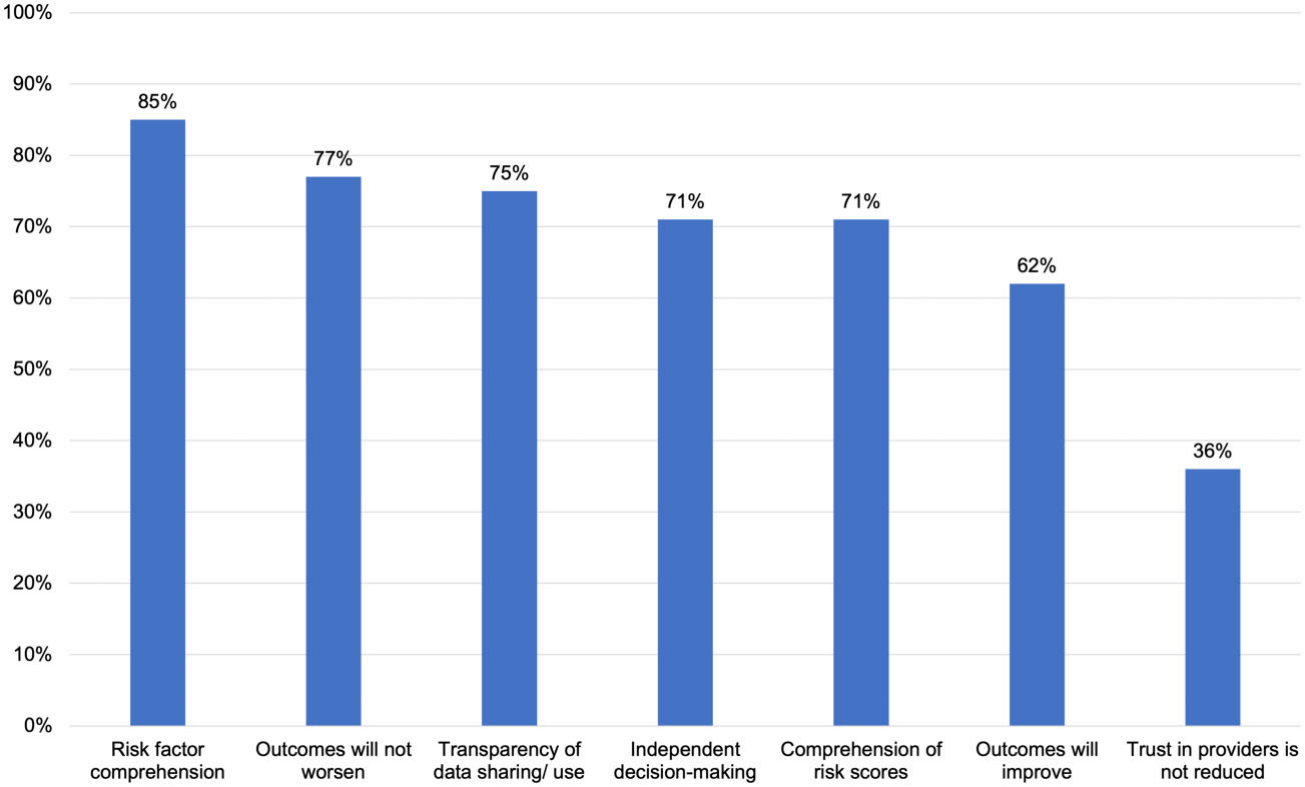
Survey respondents who endorsed each bioethical concept as “very important” as it pertains to AI in mental health care (*n* = 258). Alt text: vertical bar chart showing which constructs related to AI survey respondents found “very important.”

The most important construct for survey respondents was the ability to independently comprehend their personal risk factors that drove AI model predictions; 85% indicated this was “very” important (Figure 3). Nearly all concepts were strongly endorsed as “very important.” The construct with the least endorsement was the impact of AI on trust in mental health providers (36%), and this was the only construct with significant differences by self-reported previous pregnancy status; 51% of those with a previous pregnancy said it was “somewhat” important versus 36% of those with no previous pregnancies (*P* = .001).

## DISCUSSION

In this survey of US women, respondents were open to the use of AI-based technologies in mental health care but expressed significant concerns about unintended medical harm (for which they hold multiple parties accountable) and inappropriate data sharing. Survey respondents’ concerns about AI correlate to perceived seriousness of the diagnosis or recommended intervention. Survey respondents expressed clear discomfort with AI disrupting their relationship with their mental health provider, which is the main source of their trust in mental healthcare. Most survey respondents want to be able to independently comprehend risk scores. Taken together, these findings underscore the importance of anticipating and mitigating potential ethical concerns that arise with the use of AI-based technologies in mental health care.

These findings align with prior studies of attitudes toward AI in women’s health and in mental healthcare. For example, related to mental healthcare generally, studies of AI-powered health chatbots have reported that acceptability decreases as stigma and severity increase,^22^ and patients value nonjudgmental interactions with chatbots, and fear less human interaction will result from chatbots’ use.^23^ Related to women’s health, studies of AI implementation in women’s health care have detected a preference for clinicians to independently interpret predictions and results.^24^^,^^25^ In 1 study, Dutch women were ambivalent about blame for medical errors resulting from AI.^24^ However in our study of US women and others of US populations,^26^ most survey respondents felt clinicians were to blame for individual errors, and that healthcare systems, developers, and the government all hold responsibility for ensuring AI safety broadly.^26^

While there were only a few comparisons for which there were significant differences based on self-reported previous pregnancy status, there is added complexity for pregnancy-capable persons in need of mental health support because both pregnancy and mental illness create vulnerability that must be considered. In pregnancy, the autonomy, harms, and benefits afforded to the perinatal patient, child, and partner must all be weighed simultaneously. Prior work has noted the ethical ramifications of data collection needed to create AI models potentially leading to feelings of inappropriate surveillance and ultimately mistrust, and disproportionate benefits distributed to White and higher socioeconomic status individuals.^27^ Additionally, the possibility that personal health data may be used for surveilling women of childbearing age in the post-Roe v. Wade era has caused alarm among many communities,^28^ highlighting the need to identify effective strategies to anticipate and mitigate ethical issues is urgently needed.

Finally, this study emphasizes the importance of considering explainable AI that supports *patients*, not just clinicians. In our study, the vast majority of survey respondents wanted to be able to understand their risk scores and personal risk factors to enable more participation in medical decision-making. Similarly, in a recent study of pregnant individuals in Spain, participants reported that their confidence would increase if AI were more explainable.^29^ This is highly consistent with reports on physician trust in AI and the need for more explainable models, but little work on supporting comprehension in patient-facing AI has been conducted to date.^7^

Limitations of this study include that the sample was recruited using an online platform, which may not generalize to those with technology, literacy, and other barriers to online survey completion. Sample recruitment was based on US census age, gender, and race distributions,^15^ leading to a majority of survey respondents being White due to over 70% of the US population identifying as White in recent census data.^30^ Thus, important perspectives from other racial/ethnic groups are limited. Differences in self-reported previous pregnancy status by age and mental health rating may explain some differences in the results; future work examining attitudes among young women specifically will be important given the higher prevalence of depression in this population.

## Supplementary Material

ooad048_Supplementary_Data

## Data Availability

The data underlying this article will be shared on reasonable request to the corresponding author.

## References

[1] Topol EJ. High-performance medicine: the convergence of human and artificial intelligence. Nat Med 2019; 25 (1): 44–56. 30617339 10.1038/s41591-018-0300-7

[2] 2020 National Survey of Drug Use and Health (NSDUH) releases. https://www.samhsa.gov/data/release/2020-national-survey-drug-use-and-health-nsduh-releases. Accessed March 30, 2023.

[3] Avin S , Belfield H, Brundage M, et al Filling gaps in trustworthy development of AI. Science 2021; 374 (6573): 1327–9. 34882478 10.1126/science.abi7176

[4] Rajpurkar P , Chen E, Banerjee O, Topol EJ. AI in health and medicine. Nat Med 2022; 28 (1): 31–8. 35058619 10.1038/s41591-021-01614-0

[5] The IEEE Global Initiative on Ethics of Autonomous and Intelligent Systems. *Ethically Aligned Design: A Vision for Prioritizing Human Well-being with Autonomous and Intelligent Systems, Version 2*. IEEE; 2017. https://standards.ieee.org/wp-content/uploads/import/documents/other/ead_v2.pdf. Accessed March 30, 2023.

[6] Diprose WK , Buist N, Hua N, Thurier Q, Shand G, Robinson R. Physician understanding, explainability, and trust in a hypothetical machine learning risk calculator. J Am Med Inform Assoc [Internet] 2020; 27 (4): 592–600. 32106285 10.1093/jamia/ocz229 PMC7647292

[7] Walsh CG , McKillop MM, Lee P, Harris JW, Simpson C, Novak LL. Risky business: a scoping review for communicating results of predictive models between providers and patients. JAMIA Open 2021; 4 (4): ooab092. 34805776 10.1093/jamiaopen/ooab092 PMC8598291

[8] Benda NC , Das LT, Abramson EL, et al “How did you get to this number?” Stakeholder needs for implementing predictive analytics: a pre-implementation qualitative study. J Am Med Inform Assoc [Internet] 2020; 27 (5): 709–16. 32159774 10.1093/jamia/ocaa021 PMC7647269

[9] Salk RH , Hyde JS, Abramson LY. Gender differences in depression in representative national samples: meta-analyses of diagnoses and symptoms. Psychol Bull 2017; 143 (8): 783–822. 28447828 10.1037/bul0000102 PMC5532074

[10] Brandon AR , Shivakumar G, Lee SC, Inrig SJ, Sadler JZ. Ethical issues in perinatal mental health research. Curr Opin Psychiatry 2009; 22 (6): 601–6. 19734786 10.1097/YCO.0b013e3283318e6f PMC2841567

[11] Gavin NI , Gaynes BN, Lohr KN, Meltzer-Brody S, Gartlehner G, Swinson T. Perinatal depression: a systematic review of prevalence and incidence. Obstet Gynecol 2005; 106 (5 Pt 1): 1071–83. 16260528 10.1097/01.AOG.0000183597.31630.db

[12] Khullar D , Casalino LP, Qian Y, Lu Y, Krumholz HM, Aneja S. Perspectives of patients about artificial intelligence in health care. JAMA Netw Open 2022; 5 (5): e2210309. 35507346 10.1001/jamanetworkopen.2022.10309 PMC9069257

[13] Armero W , Gray KJ, Fields KG, Cole NM, Bates DW, Kovacheva VP. A survey of pregnant patients’ perspectives on the implementation of artificial intelligence in clinical care. J Am Med Inform Assoc 2022; 30 (1): 46–53. 36250788 10.1093/jamia/ocac200 PMC9748543

[14] Palan S , Schitter C. Prolific.ac—a subject pool for online experiments. J Behav Exp Finance 2018; 17: 22–7.

[15] Prolific’s best practice guide. https://researcher-help.prolific.co/hc/en-gb/articles/360034677314-Quick-guide-to-using-Prolific. Accessed March 30, 2023.

[16] EL Skopac JS , Mbawuike SU, Levin AT, Dwyer SJ, Rosenthal AS, Humphreys JG. *An Ethical Framework for the Use of Consumer-Generated Data in Health Care*; 2019. https://www.mitre.org/news-insights/publication/ethical-framework-use-consumer-generated-data-health-care. Accessed March 30, 2023.

[17] Doraiswamy PM , Blease C, Bodner K. Artificial intelligence and the future of psychiatry: Insights from a global physician survey. Artif Intell Med 2020; 102: 101753. 31980092 10.1016/j.artmed.2019.101753

[18] Scott KA , Britton L, McLemore MR. The ethics of perinatal care for black women: dismantling the structural racism in “mother blame” narratives. J Perinat Neonatal Nurs 2019; 33 (2): 108–15. 31021935 10.1097/JPN.0000000000000394

[19] Chew LD , Bradley KA, Boyko EJ. Brief questions to identify patients with inadequate health literacy. Fam Med 2004; 36 (8): 588–94. 15343421

[20] McNaughton CD , Cavanaugh KL, Kripalani S, Rothman RL, Wallston KA. Validation of a short, 3-item version of the subjective numeracy scale. Med Decis Making 2015; 35 (8): 932–6. 25878195 10.1177/0272989X15581800 PMC4592371

[21] Degner LF , Sloan JA, Venkatesh P. The control preferences scale. Can J Nurs Res 1997; 29 (3): 21–43. 9505581

[22] Miles O , West R, Nadarzynski T. Health chatbots acceptability moderated by perceived stigma and severity: A cross-sectional survey. Digit Health 2021; 7: 20552076211063012. 34917391 10.1177/20552076211063012 PMC8670785

[23] Nadarzynski T , Puentes V, Pawlak I, et al Barriers and facilitators to engagement with artificial intelligence (AI)-based chatbots for sexual and reproductive health advice: a qualitative analysis. Sex Health 2021; 18 (5): 385–93. 34782055 10.1071/SH21123

[24] Ongena YP , Yakar D, Haan M, Kwee TC. Artificial intelligence in screening mammography: a population survey of women’s preferences. J Am Coll Radiol 2021; 18 (1 Pt A): 79–86. 33058789 10.1016/j.jacr.2020.09.042

[25] Pesapane F , Rotili A, Valconi E, et al Women’s perceptions and attitudes to the use of AI in breast cancer screening: a survey in a cancer referral centre. Br J Radiol 2023; 96 (1141): 20220569. 36314388 10.1259/bjr.20220569 PMC11864346

[26] Khullar D , Casalino LP, Qian Y, Lu Y, Chang E, Aneja S. Public vs physician views of liability for artificial intelligence in health care. J Am Med Inform Assoc 2021; 28 (7): 1574–7. 33871009 10.1093/jamia/ocab055 PMC8279784

[27] Feldman N , Perret S. Digital mental health for postpartum women: perils, pitfalls, and promise. npj Digit Med 2023; 6 (1): 1–4. 36702915 10.1038/s41746-023-00756-4 PMC9879244

[28] Dong Z , Wang L, Xie H, Xu G, Wang H. Privacy analysis of period tracking mobile apps in the post-Roe v. Wade era. In: proceedings of the 37th IEEE/ACM International Conference on Automated Software Engineering (ASE ’22). New York, NY, USA: Association for Computing Machinery; 2023: 1–6.

[29] Oprescu AM , Miró-Amarante G, García-Díaz L, et al Towards a data collection methodology for responsible artificial intelligence in health: a prospective and qualitative study in pregnancy. Inf Fusion 2022; 83–84: 53–78.

[30] US Census Bureau. 2020 Census Illuminates Racial and Ethnic Composition of the Country, Jun 10, 2022. https://www.census.gov/library/stories/2021/08/improved-race-ethnicity-measures-reveal-united-states-population-much-more-multiracial.html. Accessed March 30, 2023.

