# Supplementary material for: Women’s perspectives on the use of artificial intelligence (AI)-based technologies in mental healthcare
Source: JAMIA Open. 2023 Jul 8;6(3):ooad048. doi: 10.1093/jamiaopen/ooad048 (PMC10329494; doi:10.1093/jamiaopen/ooad048)
Supplement: ooad048_Supplementary_Data [file ooad048_supplementary_data.zip › Supplementary File 2.pdf]

SUPPLEMENTARY FILE 2: DETAILED SURVEY RESULTS

Table 1. Demographic and clinical characteristics of participants reporting female sex at birth

	**Overall** (n=258)	**Previously pregnant** (n=140)	**Not previously pregnant** (n=118)	**p-value***
**Age** (Median, IQR)	48 (32, 60)	56 (43, 61)	35 (24, 51)	**<0.001**
**Race**				0.62
Asian	12 (4.7%)	6 (4.3%)	6 (5.1%)	
Black or African American	35 (14%)	20 (14%)	15 (13%)	
White	200 (78%)	106 (76%)	94 (80%)	
Other/Prefer not to answer	11 (4.3%)	8 (5.7%)	3 (2.5%)	
**Ethnicity**				0.62
Hispanic/Latino	17 (6.6%)	8 (5.7%)	9 (7.6%)	
Not Hispanic/Latino	241 (93%)	132 (94%)	109 (92%)	
Other/Prefer not to answer	0 (0%)	0 (0%)	0 (0%)	
**Education**				0.57
Less than Bachelor's Degree	121 (47%)	69 (49%)	52 (44%)	
Bachelor's Degree	93 (36%)	50 (36%)	43 (36%)	
More than Bachelor's Degree	44 (17%)	21 (15%)	23 (19%)	
Prefer not to answer	0 (0%)	0 (0%)	0 (0%)	
**Health Literacy**				0.31
Adequate	196 (76%)	110 (79%)	86 (73%)	
Inadequate	62 (24%)	30 (21%)	32 (27%)	
**Subjective Numeracy (Categorized)**				0.70
Low Subjective Numeracy	162 (63%)	86 (62%)	76 (64%)	
High Subjective Numeracy	95 (37%)	53 (38%)	42 (36%)	
(Missing)	1	1	0	
**Control Preferences Scale**				0.92
Make the final selection about which treatment I will receive	24 (9.3%)	14 (10%)	10 (8.5%)	
Make the final selection after seriously considering my doctor's opinion	114 (44%)	62 (44%)	52 (44%)	
Have my doctor and I share responsibility for deciding what treatment is best	101 (39%)	55 (39%)	46 (39%)	
Have my doctor make the final decision but consider my opinion	19 (7.4%)	9 (6.4%)	10 (8.5%)	
Leave all decisions regarding treatment to my doctor	0 (0%)	0 (0%)	0 (0%)	
Prefer not to answer	0 (0%)	0 (0%)	0 (0%)	
**Ever been told have mental illness**				0.052
Yes	143 (55%)	68 (49%)	75 (64%)	
No	110 (43%)	69 (49%)	41 (35%)	
Prefer not to answer	5 (1.9%)	3 (2.1%)	2 (1.7%)	
**Overall mental health rating**				**0.015**
Excellent	23 (8.9%)	17 (12%)	6 (5.1%)	
Very good	67 (26%)	44 (31%)	23 (19%)	
Good	84 (33%)	37 (26%)	47 (40%)	
Fair	62 (24%)	34 (24%)	28 (24%)	
Poor	20 (7.8%)	7 (5.0%)	13 (11%)	
Don't know	2 (0.8%)	1 (0.7%)	1 (0.8%)	

*Wilcoxon rank sum test; Fisher's Exact Test for Count Data with simulated p-value (based on 2000 replicates). Bold indicates significance at $p < 0.05$.

**Table 2.** Pregnancy-related characteristics of participants reporting female sex at birth (n=258)	
**Currently pregnant**	
Yes	2 (0.8%)
No	254 (98%)
Prefer not to answer	2 (0.8%)
**If yes, how far along in months**	
2	1 (50%)
4	1 (50%)
**Previously pregnant (not including current pregnancy)**	140 (54%)
**If yes, number of times**	
One	40 (29%)
Two	46 (33%)
Three	32 (23%)
Four or more	22 (16%)
(Missing)	118
**If yes, years since last child was born**	
Median (IQR)	21 (11, 32)
Mean (SD)	22 (13)
Range	0, 61
(Missing)	133
**Diagnosed with postpartum depression**	
Yes	15 (5.8%)
No	142 (55%)
N/A - was never pregnant	100 (39%)
Prefer not to answer	1 (0.4%)
**Ever treated for PPD**	
Yes	10 (3.9%)
No	145 (56%)
N/A - was never pregnant	102 (40%)
Prefer not to answer	1 (0.4%)

**Table 3:** General attitudes and comfort with AI being used in aspects of mental health care				
	**Overall** (n=258)	**Previously pregnant** (n=140)	**Not previously pregnant** (n=118)	**p-value***
**How much do you know about AI and how it could change mental healthcare?**				**0.02**
I know quite a lot	1 (0.4%)	1 (0.7%)	0 (0%)	
I know a fair amount	27 (10%)	18 (13%)	9 (7.6%)	
I know a little bit	137 (53%)	63 (45%)	74 (63%)	
I know almost nothing	93 (36%)	58 (41%)	35 (30%)	
**Overall, in the next 5 years, do you think AI will make mental healthcare in the United States?**				0.40
Much better	11 (4.3%)	9 (6.4%)	2 (1.7%)	
Somewhat better	104 (40%)	54 (39%)	50 (42%)	
Minimal change	89 (34%)	45 (32%)	44 (37%)	
Somewhat worse	21 (8.1%)	13 (9.3%)	8 (6.8%)	
Much worse	4 (1.6%)	3 (2.1%)	1 (0.8%)	
Don't know	29 (11%)	16 (11%)	13 (11%)	
**How comfortable: AI, instead of a mental health professional, performing a mental health assessment?**				0.23
Very comfortable	22 (8.5%)	15 (11%)	7 (5.9%)	
Somewhat comfortable	100 (39%)	51 (36%)	49 (42%)	
Somewhat uncomfortable	69 (27%)	33 (24%)	36 (31%)	
Very uncomfortable	64 (25%)	40 (29%)	24 (20%)	
Don't know	3 (1.2%)	1 (0.7%)	2 (1.7%)	
**How comfortable: AI, instead of a mental health professional, making a diagnosis of clinical depression?**				0.2
Very comfortable	13 (5.0%)	9 (6.4%)	4 (3.4%)	
Somewhat comfortable	58 (22%)	34 (24%)	24 (20%)	
Somewhat uncomfortable	82 (32%)	36 (26%)	46 (39%)	
Very uncomfortable	102 (40%)	59 (42%)	43 (36%)	
Don't know	3 (1.2%)	2 (1.4%)	1 (0.8%)	
**How comfortable: AI, instead of a mental health professional, telling you that you are clinically depressed?**				0.62
Very comfortable	12 (4.7%)	7 (5.0%)	5 (4.2%)	
Somewhat comfortable	63 (24%)	35 (25%)	28 (24%)	
Somewhat uncomfortable	71 (28%)	33 (24%)	38 (32%)	
Very uncomfortable	106 (41%)	61 (44%)	45 (38%)	
Don't know	6 (2.3%)	4 (2.9%)	2 (1.7%)	
**How comfortable: AI, instead of a mental health professional, making a diagnosis of bi-polar disorder?**				0.86
Very comfortable	13 (5.0%)	8 (5.7%)	5 (4.2%)	
Somewhat comfortable	38 (15%)	22 (16%)	16 (14%)	
Somewhat uncomfortable	75 (29%)	38 (27%)	37 (31%)	
Very uncomfortable	128 (50%)	69 (49%)	59 (50%)	
Don't know	4 (1.6%)	3 (2.1%)	1 (0.8%)	

**How comfortable: AI, instead of a mental health professional, telling you that you directly that you have bi-polar disorder?**				0.21
Very comfortable	11 (4.3%)	6 (4.3%)	5 (4.2%)	
Somewhat comfortable	32 (12%)	20 (14%)	12 (10%)	
Somewhat uncomfortable	78 (30%)	35 (25%)	43 (36%)	
Very uncomfortable	133 (52%)	78 (56%)	55 (47%)	
Don't know	4 (1.6%)	1 (0.7%)	3 (2.5%)	
**How comfortable: AI, instead of a mental health professional, recommending a general wellness or stress-management strategy?**				0.27
Very comfortable	76 (29%)	41 (29%)	35 (30%)	
Somewhat comfortable	111 (43%)	63 (45%)	48 (41%)	
Somewhat uncomfortable	41 (16%)	18 (13%)	23 (19%)	
Very uncomfortable	28 (11%)	18 (13%)	10 (8.5%)	
Don't know	2 (0.8%)	0 (0%)	2 (1.7%)	
**How comfortable: AI, instead of a mental health professional, recommending a talk therapy?**				0.48
Very comfortable	62 (24%)	37 (26%)	25 (21%)	
Somewhat comfortable	106 (41%)	56 (40%)	50 (42%)	
Somewhat uncomfortable	41 (16%)	20 (14%)	21 (18%)	
Very uncomfortable	42 (16%)	25 (18%)	17 (14%)	
Don't know	7 (2.7%)	2 (1.4%)	5 (4.2%)	
**How comfortable: AI, instead of a mental health professional, recommending a medication?**				0.93
Very comfortable	13 (5.0%)	7 (5.0%)	6 (5.1%)	
Somewhat comfortable	49 (19%)	24 (17%)	25 (21%)	
Somewhat uncomfortable	79 (31%)	43 (31%)	36 (31%)	
Very uncomfortable	115 (45%)	65 (46%)	50 (42%)	
Don't know	2 (0.8%)	1 (0.7%)	1 (0.8%)	
**How comfortable: AI, instead of a mental health professional, predicting a patient's risk for suicide?**				0.79
Very comfortable	19 (7.4%)	12 (8.6%)	7 (5.9%)	
Somewhat comfortable	55 (21%)	27 (19%)	28 (24%)	
Somewhat uncomfortable	67 (26%)	35 (25%)	32 (27%)	
Very uncomfortable	105 (41%)	60 (43%)	45 (38%)	
Don't know	12 (4.7%)	6 (4.3%)	6 (5.1%)	
**How comfortable: AI, instead of a mental health professional, predicting a patient's risk of engaging in violent behavior?**				0.39
Very comfortable	20 (7.8%)	13 (9.3%)	7 (5.9%)	
Somewhat comfortable	58 (22%)	32 (23%)	26 (22%)	
Somewhat uncomfortable	62 (24%)	28 (20%)	34 (29%)	
Very uncomfortable	105 (41%)	61 (44%)	44 (37%)	
Don't know	13 (5.0%)	6 (4.3%)	7 (5.9%)	

*Wilcoxon rank sum test; Fisher's Exact Test for Count Data with simulated p-value (based on 2000 replicates). Bold indicates significance at $p < 0.05$.

**Table 4:** Levels of specific concerns regarding the use of AI in mental health care				
	**Overall** (n=258)	**Previously pregnant** (n=140)	**Not previously pregnant** (n=118)	**p-value***
**That my mental health information will not be kept confidential**				0.39
Very concerned	55 (21%)	30 (21%)	25 (21%)	
Somewhat concerned	100 (39%)	50 (36%)	50 (42%)	
Not concerned	100 (39%)	57 (41%)	43 (36%)	
Don't know	3 (1.2%)	3 (2.1%)	0 (0%)	
**That the AI will make the wrong diagnosis about my mental health**				0.11
Very concerned	125 (49%)	71 (51%)	54 (46%)	
Somewhat concerned	109 (42%)	52 (37%)	57 (48%)	
Not concerned	23 (8.9%)	16 (12%)	7 (5.9%)	
Don't know	0 (0%)	0 (0%)	0 (0%)	
(Missing)	1	1	0	
**That the AI will lead to me getting inappropriate treatment for my mental health**				0.19
Very concerned	130 (50%)	75 (54%)	55 (47%)	
Somewhat concerned	94 (36%)	44 (31%)	50 (42%)	
Not concerned	32 (12%)	19 (14%)	13 (11%)	
Don't know	2 (0.8%)	2 (1.4%)	0 (0%)	
**That AI will mean I spend less time with my mental health professional**				**0.032**
Very concerned	108 (42%)	54 (39%)	54 (46%)	
Somewhat concerned	74 (29%)	37 (26%)	37 (31%)	
Not concerned	65 (25%)	45 (32%)	20 (17%)	
Don't know	11 (4.3%)	4 (2.9%)	7 (5.9%)	
**That AI will lead to my mental health provider not knowing me as well**				0.3
Very concerned	129 (50%)	64 (46%)	65 (55%)	
Somewhat concerned	89 (34%)	50 (36%)	39 (33%)	
Not concerned	36 (14%)	24 (17%)	12 (10%)	
Don't know	4 (1.6%)	2 (1.4%)	2 (1.7%)	
**That AI will increase my mental health care costs**				0.17
Very concerned	47 (18%)	29 (21%)	18 (15%)	
Somewhat concerned	68 (26%)	32 (23%)	36 (31%)	
Not concerned	121 (47%)	63 (45%)	58 (49%)	
Don't know	21 (8.2%)	15 (11%)	6 (5.1%)	
(Missing)	1	1	0	
*Fisher's Exact Test for Count Data with simulated p-value (based on 2000 replicates); Fisher's Exact Test for Count Data				

Table 5: Comfort with sharing personal health information and AI being used in specific scenarios				
	**Overall** (n=258)	**Previously pregnant** (n=140)	**Not previously pregnant** (n=118)	**p-value***
**Comfort sharing private information with a human mental health professional**				0.58
Very comfortable	101 (39%)	57 (41%)	44 (37%)	
Somewhat comfortable	105 (41%)	56 (40%)	49 (42%)	
Somewhat uncomfortable	42 (16%)	20 (14%)	22 (19%)	
Very uncomfortable	10 (3.9%)	7 (5.0%)	3 (2.5%)	
Don't know	0 (0%)	0 (0%)	0 (0%)	
**Comfort sharing private information with an AI chatbot**				0.96
Very comfortable	41 (16%)	23 (16%)	18 (15%)	
Somewhat comfortable	83 (32%)	44 (31%)	39 (33%)	
Somewhat uncomfortable	66 (26%)	34 (24%)	32 (27%)	
Very uncomfortable	64 (25%)	37 (26%)	27 (23%)	
Don't know	4 (1.6%)	2 (1.4%)	2 (1.7%)	
**Comfort sharing private information to help improve AI programs that treat disease**				0.63
Very comfortable	56 (22%)	28 (20%)	28 (24%)	
Somewhat comfortable	103 (40%)	60 (43%)	43 (36%)	
Somewhat uncomfortable	52 (20%)	26 (19%)	26 (22%)	
Very uncomfortable	32 (12%)	16 (11%)	16 (14%)	
Don't know	15 (5.8%)	10 (7.1%)	5 (4.2%)	
**How important do you think it is that you are told when an AI program has played a big role in your mental health diagnosis or treatment?**				0.14
Not important	7 (2.7%)	1 (0.7%)	6 (5.1%)	
Somewhat important	45 (17%)	27 (19%)	18 (15%)	
Very important	199 (77%)	109 (78%)	90 (76%)	
Don't know	7 (2.7%)	3 (2.1%)	4 (3.4%)	
**How important do you think it is that you are told when an AI program has played a small role in your mental health diagnosis or treatment?**				**0.026**
Not important	15 (5.8%)	5 (3.6%)	10 (8.5%)	
Somewhat important	102 (40%)	50 (36%)	52 (44%)	
Very important	134 (52%)	83 (59%)	51 (43%)	
Don't know	7 (2.7%)	2 (1.4%)	5 (4.2%)	
**Depression scenario (see below**): How does the computer program affect your view?**				0.59
Not important	14 (5.4%)	6 (4.3%)	8 (6.8%)	
Somewhat important	59 (23%)	29 (21%)	30 (25%)	
Very important	182 (71%)	103 (74%)	79 (67%)	
Don't know	3 (1.2%)	2 (1.4%)	1 (0.8%)	
**How comfortable would you be receiving a mental health diagnosis from a computer program that made the right diagnosis 90% of the time but could not explain why it made the diagnosis?**				0.9

Very comfortable	7 (2.7%)	4 (2.9%)	3 (2.5%)	
Somewhat comfortable	46 (18%)	26 (19%)	20 (17%)	
Somewhat uncomfortable	95 (37%)	54 (39%)	41 (35%)	
Very uncomfortable	105 (41%)	53 (38%)	52 (44%)	
Don't know	5 (1.9%)	3 (2.1%)	2 (1.7%)	
**How comfortable would you be receiving a mental health diagnosis from a computer program that made the right diagnosis 98% of the time but could not explain why it made the diagnosis?**				0.87
Very comfortable	29 (11%)	16 (11%)	13 (11%)	
Somewhat comfortable	70 (27%)	35 (25%)	35 (30%)	
Somewhat uncomfortable	82 (32%)	47 (34%)	35 (30%)	
Very uncomfortable	70 (27%)	39 (28%)	31 (26%)	
Don't know	7 (2.7%)	3 (2.1%)	4 (3.4%)	
**Symptoms scenario (see below***): How does the computer program affect your view?**				**0.007**
It would not affect my trust of the mental health professional's assessment	39 (15%)	28 (20%)	11 (9.3%)	
It would make me question the mental health professional's assessment	137 (53%)	69 (49%)	68 (58%)	
I do not know if it would change my view of the mental health professional's assessment	76 (29%)	37 (26%)	39 (33%)	
Don't know	6 (2.3%)	6 (4.3%)	0 (0%)	

*Wilcoxon rank sum test; Fisher's Exact Test for Count Data with simulated p-value (based on 2000 replicates). Bold indicates significance at $p < 0.05$.

**Depression scenario: Imagine that you have been told that you have been diagnosed with depression, a common mental illness that affects your mood, thoughts, and behavior. In the past, your doctor would have decided whether to prescribe a medication or refer you for psychotherapy depending on the type of symptoms you have and how severe they are. Your doctor now has a computer program that uses many other factors. This computer program says you should start an antidepressant. How important is it that your doctor tells you that the computer program helped make this decision?

***Symptom scenario: Imagine that you have some symptoms that have been bothering you for a while, such as difficulty sleeping, eating, and focusing on work. You visit a doctor who runs some tests and he says he does NOT think you have any mental health issue. He also puts your symptoms into a computer program that can make the right diagnosis about 80% of the time, but can't say why it chose the diagnoses. It says you DO have mental health issue. How does the computer program affect your view?

**Table 6:** Perceptions of responsible parties for AI use in mental health care				
	**Overall** (n=258)	**Previously pregnant** (n=140)	**Not previously pregnant** (n=118)	**p-value***
**Imagine that your mental health professional and a computer program work together to treat your mental illness and a medical error occurs. An example of a medical error is getting a diagnosis that was wrong, or a treatment that was not needed. Who is responsible?**				
Mental health professional	215 (83%)	121 (86%)	94 (80%)	0.18
Company that made the computer program	87 (34%)	46 (33%)	41 (35%)	0.79
Hospital or clinic that bought the computer program	73 (28%)	37 (26%)	36 (31%)	0.49
Government agency that approved the computer program	55 (21%)	28 (20%)	27 (23%)	0.65
Someone else	11 (4.3%)	4 (2.9%)	7 (5.9%)	0.35
No one	1 (0.4%)	0 (0%)	1 (0.8%)	0.46
Don't know	17 (6.6%)	7 (5.0%)	10 (8.5%)	0.32
**Imagine that you have a sleeping disorder that might be due to a mental health issue. You have a test done. Your doctor uses a computer program that says the sleeping disorder might be mental health-related, so you start medication to treat it. The medication leads to bad side effects. After another doctor evaluates your sleeping disorder, it turns out it was NOT mental health-related. Who, if anyone, is to blame?**				
Mental health professional	212 (82%)	119 (85%)	93 (79%)	0.25
Company that made the computer program	78 (30%)	41 (29%)	37 (31%)	0.79
Hospital or clinic that bought the computer program	57 (22%)	29 (21%)	28 (24%)	0.65
Government agency that approved the computer program	49 (19%)	25 (18%)	24 (20%)	0.64
Someone else	6 (2.3%)	3 (2.1%)	3 (2.5%)	>0.99
No one	10 (3.9%)	4 (2.9%)	6 (5.1%)	0.52
Don't know	9 (3.5%)	4 (2.9%)	5 (4.2%)	0.74
**Imagine that your hospital recently started using a computer program to help diagnose mental health problems. Who do you think has checked to make sure the computer program is safe before it is rolled out?**				
Mental health professional	61 (24%)	36 (26%)	25 (21%)	0.46
Company that made the computer program	171 (66%)	92 (66%)	79 (67%)	0.9
Hospital or clinic that bought the computer program	149 (58%)	78 (56%)	71 (60%)	0.53
Government agency that approved the computer program	122 (47%)	57 (41%)	65 (55%)	**0.024**
Someone else	4 (1.6%)	2 (1.4%)	2 (1.7%)	>0.99
No one	9 (3.5%)	6 (4.3%)	3 (2.5%)	0.51
Don't know	6 (2.3%)	3 (2.1%)	3 (2.5%)	>0.99
*Wilcoxon rank sum test; Fisher's Exact Test for Count Data with simulated p-value (based on 2000 replicates). Bold indicates significance at $p < 0.05$.				

**Table 7:** Importance of specific bioethical constructs in general, for other people				
	**Overall** (n=258)	**Previously pregnant** (n=140)	**Not previously pregnant** (n=118)	**p-value***
**That people are able to make up their own mind about their risk for depression based on AI output**				0.67
Very important	152 (59%)	84 (60%)	68 (58%)	
Somewhat important	84 (33%)	43 (31%)	41 (35%)	
Not important	10 (3.9%)	7 (5.0%)	3 (2.5%)	
Don't know	12 (4.7%)	6 (4.3%)	6 (5.1%)	
**That AI will improve depressive symptoms**				0.74
Very important	112 (43%)	65 (46%)	47 (40%)	
Somewhat important	103 (40%)	52 (37%)	51 (43%)	
Not important	19 (7.4%)	10 (7.1%)	9 (7.6%)	
Don't know	24 (9.3%)	13 (9.3%)	11 (9.3%)	
**That AI will reduce the chance of negative outcomes**				0.93
Very important	193 (75%)	104 (74%)	89 (75%)	
Somewhat important	54 (21%)	29 (21%)	25 (21%)	
Not important	7 (2.7%)	4 (2.9%)	3 (2.5%)	
Don't know	4 (1.6%)	3 (2.1%)	1 (0.8%)	
**That people can understand how likely it is that they develop depression in the next year according to the AI**				0.96
Very important	172 (67%)	93 (66%)	79 (67%)	
Somewhat important	65 (25%)	35 (25%)	30 (25%)	
Not important	6 (2.3%)	4 (2.9%)	2 (1.7%)	
Don't know	15 (5.8%)	8 (5.7%)	7 (5.9%)	
**That AI does not reduce people's trust in their mental health care professionals**				0.088
Very important	76 (29%)	42 (30%)	34 (29%)	
Somewhat important	124 (48%)	73 (52%)	51 (43%)	
Not important	37 (14%)	13 (9.3%)	24 (20%)	
Don't know	21 (8.1%)	12 (8.6%)	9 (7.6%)	
**That people are aware of how their personal data is being used for AI**				0.56
Very important	199 (77%)	104 (74%)	95 (81%)	
Somewhat important	45 (17%)	26 (19%)	19 (16%)	
Not important	7 (2.7%)	5 (3.6%)	2 (1.7%)	
Don't know	7 (2.7%)	5 (3.6%)	2 (1.7%)	
**That people can understand which of their individual risk factors for depression are used by the AI**				0.48
Very important	230 (89%)	122 (87%)	108 (92%)	
Somewhat important	20 (7.8%)	12 (8.6%)	8 (6.8%)	
Not important	3 (1.2%)	3 (2.1%)	0 (0%)	
Don't know	5 (1.9%)	3 (2.1%)	2 (1.7%)	
*Wilcoxon rank sum test; Fisher's Exact Test for Count Data with simulated p-value (based on 2000 replicates). Bold indicates significance at $p < 0.05$.				

**Table 8:** Importance of specific bioethical constructs for the individual participant				
	**Overall** (n=258)	**Previously pregnant** (n=140)	**Not previously pregnant** (n=118)	**p-value***
**That you are able to make up your own mind about your risk for depression based on AI output**				0.69
Very important	182 (71%)	97 (69%)	85 (72%)	
Somewhat important	61 (24%)	36 (26%)	25 (21%)	
Not important	7 (2.7%)	4 (2.9%)	3 (2.5%)	
Don't know	8 (3.1%)	3 (2.1%)	5 (4.2%)	
**That AI will improve your depression/ depressive symptoms**				0.17
Very important	159 (62%)	90 (64%)	69 (58%)	
Somewhat important	67 (26%)	31 (22%)	36 (31%)	
Not important	18 (7.0%)	13 (9.3%)	5 (4.2%)	
Don't know	14 (5.4%)	6 (4.3%)	8 (6.8%)	
**That AI will decrease the chance of negative outcomes**				0.5
Very important	198 (77%)	104 (74%)	94 (80%)	
Somewhat important	52 (20%)	31 (22%)	21 (18%)	
Not important	5 (1.9%)	4 (2.9%)	1 (0.8%)	
Don't know	3 (1.2%)	1 (0.7%)	2 (1.7%)	
**That you can understand how likely it is that you develop depression within the next year according to the AI**				0.62
Very important	182 (71%)	101 (72%)	81 (69%)	
Somewhat important	57 (22%)	27 (19%)	30 (25%)	
Not important	6 (2.3%)	4 (2.9%)	2 (1.7%)	
Don't know	13 (5.0%)	8 (5.7%)	5 (4.2%)	
**That using AI does not reduce your trust in your mental health care provider**				**0.001**
Very important	92 (36%)	46 (33%)	46 (39%)	
Somewhat important	115 (45%)	72 (51%)	43 (36%)	
Not important	31 (12%)	8 (5.7%)	23 (19%)	
Don't know	20 (7.8%)	14 (10%)	6 (5.1%)	
**That you are aware of how your personal data is being used for AI**				0.14
Very important	193 (75%)	98 (70%)	95 (81%)	
Somewhat important	47 (18%)	28 (20%)	19 (16%)	
Not important	14 (5.4%)	11 (7.9%)	3 (2.5%)	
Don't know	4 (1.6%)	3 (2.1%)	1 (0.8%)	
**That you can understand which of your individual risk factors for depression are used by the AI**				0.72
Very important	220 (85%)	117 (84%)	103 (87%)	
Somewhat important	31 (12%)	18 (13%)	13 (11%)	
Not important	5 (1.9%)	4 (2.9%)	1 (0.8%)	
Don't know	2 (0.8%)	1 (0.7%)	1 (0.8%)	
*Wilcoxon rank sum test; Fisher's Exact Test for Count Data with simulated p-value (based on 2000 replicates). Bold indicates significance at p < 0.05.				
