# Supplementary material for: Women’s perspectives on the use of artificial intelligence (AI)-based technologies in mental healthcare
Source: JAMIA Open. 2023 Jul 8;6(3):ooad048. doi: 10.1093/jamiaopen/ooad048 (PMC10329494; doi:10.1093/jamiaopen/ooad048)
Supplement: ooad048_Supplementary_Data [file ooad048_supplementary_data.zip › Supplementary File 1.pdf]

IRIS Patient facing AI: Prolific study #1

Start of Block: Consent Form

consent_sheet **Please view the information sheet using the link below before proceeding.**

consent_q By clicking “yes” you are indicating that you voluntarily agree to participate in this study. Note: if you click "no" the survey will end now.

- Yes, I consent. (1)
- No, I do not consent (survey will end). (4)

future_data_use By checking “Yes” and signing this consent form, you agree to give your survey data, including Protected Health Information, to Weill Cornell Medicine and/or Iris OB Health for research purposes.

- YES, I give permission for my survey data to be stored for future unspecified research by the researchers of this study. I understand that the data and/or samples will be stored indefinitely. (1)
- YES, I give permission for my survey and focus group data to be **shared** with other qualified **researchers** for future research. (2)
- **NO,** I do not give permission for my survey data to be stored or shared for future research. (4)

End of Block: Consent Form

Start of Block: Part 1 AI for Mental Health

Text1 **Helpful definitions:**

Artificial intelligence, known as "AI", is now being used in some parts of health care. AI usually refers to computer programs that can copy human intelligence and learn automatically. AI is being tested to see if computer programs can do things like read brain scans, assess risk, predict adverse health outcomes, offer treatment algorithms, and support medical decision making.

Mental health includes our emotional, psychological, and social well-being. It affects how we think, feel, and act. Mental healthcare is any health care service for your mental health, and could be delivered by professionals in psychology, psychiatry, neurology, social work, and other specialties. It is sometimes referred to as behavioral health care.

Clinical depression is a common mood disorder in which feelings of sadness, loss, anger, or frustration interfere with everyday life for weeks or more.

Bipolar disorder is a mood disorder that can cause intense mood swings. Along with the mood swings, bipolar disorder causes changes in behavior, energy levels, and activity levels. Bipolar disorder used to be called other names, including manic depression and manic-depressive disorder.

Trust1 How much do you know about Artificial Intelligence (AI) and how it could change mental healthcare?

○ I know quite a lot (1)

○ I know a fair amount (2)

○ I know a little bit (3)

○ I know almost nothing (4)

Page Break

Trust2 Overall, in the next 5 years, do you think AI will make mental healthcare in the United States:

- ○ Much better (1)
- ○ Somewhat better (2)
- ○ Minimal change (3)
- ○ Somewhat worse (4)
- ○ Much worse (5)
- ○ Don't know (6)

Page Break

Trust3 For each of the following, how comfortable would you feel with AI doing some of the things a mental health professional usually does?

	Very comfortable (1)	Somewhat comfortable (2)	Somewhat uncomfortable (3)	Very uncomfortable (4)	Don’t know (5)
AI, instead of a mental health professional, performing a mental health assessment? (1)	o	o	o	o	o
AI, instead of a mental health professional, making a diagnosis of clinical depression*? (2)	o	o	o	o	o
AI, instead of a mental health professional, telling you that you are clinically depressed? (3)	o	o	o	o	o
AI, instead of a mental health professional, making a diagnosis of bi-polar disorder**? (4)	o	o	o	o	o
AI, instead of a mental health professional, telling you that you directly that you have bi-polar disorder? (5)	o	o	o	o	o

AI, instead of a mental health professional, recommending a general wellness or stress-management strategy (e.g. deep breathing, going for a walk)? (6)	○	○	○	○	○
AI, instead of a mental health professional, recommending a talk therapy? (7)	○	○	○	○	○
AI, instead of a mental health professional, recommending a medication? (8)	○	○	○	○	○
AI, instead of a mental health professional, predicting a patient’s risk for suicide? (9)	○	○	○	○	○
AI, instead of a mental health professional, predicting a patient’s risk of engaging in violent behavior? (10)	○	○	○	○	○

Text2 DEFINITIONS:
*Clinical depression is a common mood disorder in which feelings of sadness, loss, anger, or frustration interfere with everyday life for weeks or more.
**Bi-polar disorder is a mood disorder that can cause intense mood swings. Along with the mood swings, bipolar disorder causes changes in behavior, energy levels, and activity levels. Bipolar disorder used to be called other names, including manic depression and manic-depressive disorder.

Page Break

Trust4 Please rate how concerned you are about the use of AI in mental health for each of the following possible reasons:

	Very concerned (1)	Somewhat concerned (2)	Not concerned (3)	Don't know (4)
That my mental health information will not be kept confidential (1)	○	○	○	○
That the AI will make the wrong diagnosis about my mental health (2)	○	○	○	○
That the AI will lead to me getting inappropriate treatment for my mental health (3)	○	○	○	○
That AI will mean I spend less time with my mental health professional (4)	○	○	○	○
That AI will lead to my mental health provider not knowing me as well (5)	○	○	○	○
That AI will increase my mental health care costs (6)	○	○	○	○

Trust5 Related to the question above, is there anything else you might be concerned about related to the use of AI for mental health scenarios? Please describe here.

____

Page Break

Trust6 Next, how comfortable would you feel sharing private information (for example, your mental health history, whether you have taken drugs, whether you have attempted suicide) in each of the following situations:

	Very comfortable (1)	Somewhat comfortable (2)	Somewhat uncomfortable (3)	Very uncomfortable (4)	Don't know (5)
With a human mental health professional (1)	○	○	○	○	○
With an AI chatbot (2)	○	○	○	○	○
To help improve AI programs that treat disease (6)	○	○	○	○	○

Trust7 How important do you think it is that you are told when an AI program has played a big role in your mental health diagnosis or treatment?

○ Not important (1)

○ Somewhat important (2)

○ Very important (3)

○ Don't know (4)

Trust8 How important do you think it is that you are told when an AI program has played a small role in your mental health diagnosis or treatment?

- ○ Not important (1)
- ○ Somewhat important (2)
- ○ Very important (3)
- ○ Don't know (4)

Trust9 Imagine that you have been told that you have been diagnosed with depression, a common mental illness that affects your mood, thoughts, and behavior. In the past, your doctor would have decided whether to prescribe a medication or refer you for psychotherapy depending on the type of symptoms you have and how severe they are.

Your doctor now has a computer program that uses many other factors. This computer program says you should start an antidepressant. How important is it that your doctor tells you that the computer program helped make this decision?

- ○ Not important (1)
- ○ Somewhat important (2)
- ○ Very important (3)
- ○ Don't know (4)

Trust10 How comfortable would you be receiving a mental health diagnosis from a computer program that made the right diagnosis 90% of the time but could not explain why it made the diagnosis?

- Very comfortable (1)
- Somewhat comfortable (2)
- Somewhat uncomfortable (3)
- Very uncomfortable (4)
- Don’t know (5)

Trust11 How comfortable would you be receiving a mental health diagnosis from a computer program that made the right diagnosis 98% of the time but could not explain why it made the diagnosis?

- Very comfortable (1)
- Somewhat comfortable (2)
- Somewhat uncomfortable (3)
- Very uncomfortable (4)
- Don’t know (5)

Attention_check1 The test you are about to take part in is very simple: When asked whether you are comfortable with public speaking, you must select "Somewhat uncomfortable." This is an attention check.

Are you comfortable with public speaking?

- ○ Very comfortable (1)
- ○ Somewhat comfortable (2)
- ○ Somewhat uncomfortable (3)
- ○ Very uncomfortable (4)
- ○ Don't know (5)

Trust12 Imagine that you have some symptoms that have been bothering you for a while, such as difficulty sleeping, eating, and focusing on work. You visit a doctor who runs some tests and he says he does NOT think you have any mental health issue. He also puts your symptoms into a computer program that can make the right diagnosis about 80% of the time, but can't say why it chose the diagnoses. It says you DO have mental health issue.

How does the computer program affect your view?

- ○ It would not affect my trust of the mental health professional's assessment. (1)
- ○ It would make me question the mental health professional's assessment. (2)
- ○ I do not know if it would change my view of the mental health professional's assessment. (3)
- ○ Don't know (4)

Trust13 Imagine that your mental health professional and a computer program work together to treat your mental illness and a medical error occurs. An example of a medical error is getting a

diagnosis that was wrong, or a treatment that was not needed. Who is responsible? (Please select all that apply.)

- ☐ The mental health professional who made the decision (1)
- ☐ The company that made the computer program (2)
- ☐ The hospital or clinic that bought the computer program (3)
- ☐ The government agency that approved the computer program (4)
- ☐ Someone else (Please specify) (5) ____
- ☐ No one (6)
- ☐ Don’t know (7)

Trust14 Imagine that you have a sleeping disorder that might be due to a mental health issue. You have a test done. Your doctor uses a computer program that says the sleeping disorder might be mental health-related, so you start medication to treat it. The medication leads to bad side effects. After another doctor evaluates your sleeping disorder, it turns out it was NOT mental health-related. Who, if anyone, is to blame?

(Please select all that apply)

- ▢ The mental health professional who made the decision (1)
- ▢ The company that made the computer program (2)
- ▢ The hospital that bought the computer program (3)
- ▢ The government agency that approved the computer program (4)
- ▢ Someone else (Please specify) (5) ____
- ▢ No one (6)
- ▢ Don't know (7)

Trust15 Imagine that your hospital recently started using a computer program to help diagnose mental health problems. Who do you think has checked to make sure the computer program is

safe before it is rolled out?
(Please select all that apply)

- ☐ The mental health professional who made the decision (1)
- ☐ The company that made the computer program (2)
- ☐ The hospital or clinic that bought the computer program (3)
- ☐ The government agency that approved the computer program (4)
- ☐ Someone else (Please specify) (5) ____
- ☐ No one (6)
- ☐ Don't know (7)

End of Block: Part 1 AI for Mental Health

Start of Block: Part 2 Bioethics

Bioethics1 As a reminder, artificial intelligence, known as "AI", is now being used in some parts of health care. AI usually refers to computer programs that can copy human intelligence and learn automatically. AI is being tested to see if computer programs can do things like read brain scans, help make mental health diagnosis, figure out when patients may be at risk for psychiatric hospitalization, and make mental health treatment recommendations. Mental health includes our emotional, psychological, and social well-being. It affects how we think, feel, and act.

Mental healthcare is any health care service for your mental health, and could be delivered by professionals in psychology, psychiatry, neurology, social work, and other specialties. It is sometimes referred to as behavioral health care.

For the following questions, we will use the mental health disorder of depression as an example to understand your thoughts related to AI and its use in mental health. Depression is a common mental illness that affects your mood, thoughts, and behavior.

Please share how important each of the following items are in general:

	Very important (1)	Somewhat important (2)	Not important (3)	Don't know (6)
That people are able to make up their own mind about their risk for depression based on AI output. (1)	○	○	○	○
That AI will improve depressive symptoms. (2)	○	○	○	○
That AI will reduce the chance of negative outcomes. (8)	○	○	○	○
That people can understand how likely it is that they develop depression in the next year according to the AI. (3)	○	○	○	○
That AI does **not** reduce people's trust in their mental health care professionals. (4)	○	○	○	○
That people are aware of how their personal data is being used for AI. (5)	○	○	○	○
That people can understand which of their individual risk factors for depression are used by the AI. (6)	○	○	○	○

Attention_check2 The test you are about to take part in is very simple: When asked how important eating breakfast is, you must select "Very important." This is an attention check.

How important is eating breakfast?

- Very important (1)
- Somewhat important (2)
- Not important (3)
- Don't know (4)

Bioethics2 Next, we again want you to imagine your hospital recently started using AI programs to support treatment for depression. Please share how important each of the following items are in terms your own mental health:

	Very important (1)	Somewhat important (2)	Not important (3)	Don't know (6)
That you are able to make up your own mind about your risk for depression based on AI output. (1)	○	○	○	○
That AI will improve your depression/depressive symptoms (2)	○	○	○	○
That AI will decrease the chance of negative outcomes. (7)	○	○	○	○
That you can understand how likely it is that you develop depression within the next year according to the AI. (3)	○	○	○	○
That using AI does **not** reduce your trust in your mental health care provider. (4)	○	○	○	○
That you are aware of how your personal data is being used for AI. (5)	○	○	○	○
That you can understand which of your individual risk factors for depression are used by the AI. (6)	○	○	○	○

Bioethics3 Please describe other ethical concerns you have about AI being used in mental health care

____

____

____

____

____

End of Block: Part 2 Bioethics

Start of Block: Health Literacy

Chew1 Thank you for your thoughts related to AI and mental health. The rest of the questions will help us learn more about you so that we can understand if we are missing any groups of people with our survey.

How often do you have problems learning about your medical condition because of difficulty understanding written information?

- ○ Never (1)
- ○ Occasionally (2)
- ○ Sometimes (3)
- ○ Often (4)
- ○ Always (5)
- ○ Prefer not to answer (6)

Chew2 How confident are you with filling out medical forms by yourself?

- Never (1)
- Occasionally (2)
- Sometimes (3)
- Often (4)
- Always (5)
- Prefer not to answer (6)

Chew3 How often do you have someone help you read hospital materials?

- Never (1)
- Occasionally (2)
- Sometimes (3)
- Often (4)
- Always (5)
- Prefer not to answer (6)

End of Block: Health Literacy

Start of Block: Numeracy

SNS1 How good are you at working with fractions?

- Not at all (1)
- Slightly (2)
- Somewhat (3)
- Moderately (4)
- Very (5)
- Extremely (6)
- Prefer not to answer (7)

SNS2 How good are you at figuring out how much a shirt will cost if it is 25% off?

- Not at all (1)
- Slightly (2)
- Somewhat (3)
- Moderately (4)
- Very (5)
- Extremely (6)
- Prefer not to answer (7)

SNS3 How often do you find numerical information useful?

- ○ Not at all (1)
- ○ Slightly (2)
- ○ Somewhat (3)
- ○ Moderately (4)
- ○ Very (5)
- ○ Extremely (6)
- ○ Prefer not to answer (7)

End of Block: Numeracy

Start of Block: Control Preferences Scale

CPS In terms of making decisions about your health care with your doctor, which ONE of the following best describes how you would like to make these decisions?

- ○ Make the final selection about which treatment I will receive (1)
- ○ Make the final selection after seriously considering my doctor's opinion (2)
- ○ Have my doctor and I share responsibility for deciding what treatment is best (3)
- ○ Have my doctor make the final decision but consider my opinion (4)
- ○ Leave all decisions regarding treatment to my doctor (5)
- ○ Prefer not to answer (6)

End of Block: Control Preferences Scale

Start of Block: Demographic questions

Sex What sex were you assigned at birth, on your original birth certificate?

- ○ Female (1)
- ○ Male (2)
- ○ Don't know (3)
- ○ Prefer not to answer (5)

Gender What is your current gender?

- ○ Female (1)
- ○ Male (2)
- ○ Transgender (3)
- ○ I use a different term (4) ____
- ○ Don't know (5)
- ○ Prefer not to answer (6)

Page Break

Age What is your age?

____

Race1 What race do you identify as? [select all that apply]

- ▢ American Indian or Alaskan Native (1)
- ▢ Asian (2)
- ▢ Black or African American (3)
- ▢ Native Hawaiian or Other Pacific Islander (4)
- ▢ White (5)
- ▢ Prefer not to answer (7)

Display This Question:

If If What race do you identify as? [select all that apply] q://QID26/SelectedChoicesCount Is Greater Than 1

Carry Forward Selected Choices from "What race do you identify as? [select all that apply]"

Race2 With which race do you identify most?

- ○ American Indian or Alaskan Native (1)
- ○ Asian (2)
- ○ Black or African American (3)
- ○ Native Hawaiian or Other Pacific Islander (4)
- ○ White (5)
- ○ Prefer not to answer (6)

Ethnicity What ethnicity do you identify as?

- ○ Hispanic/Latino (1)
- ○ Not Hispanic/Latino (2)
- ○ Unsure/prefer not to answer (3)

Country What is your country of origin?

____

____

____

____

____

Language Do you speak a language other than English in your home?

- ○ Yes (1)
- ○ No (2)
- ○ Unsure/prefer not to answer (3)

Finances Do you feel you have enough financial resources to make ends meet?

- ○ More than enough (1)
- ○ Enough (2)
- ○ Not enough (3)
- ○ Prefer not to answer (4)

Education What is the highest level of education you completed?

- ○ Did not complete high school or receive GED (8)
- ○ High school diploma or GED (9)
- ○ Some college or Associates degree (for example: AA, AS) (12)
- ○ Bachelor's degree (for example: BA. BS) (14)
- ○ Master's degree (for example: MA, MS, MEng, MEd, MSW, MBA) (15)
- ○ Professional degree beyond bachelor's degree (for example: MD, DDS, DVM, LLB, JD) (16)
- ○ Doctorate degree (for example, PhD, EdD) (17)
- ○ Prefer not to answer (18)

End of Block: Demographic questions

Start of Block: Mental health history and symptom questions

Text3 Now we are going to ask you some questions about your mental health. If you do not wish to answer certain questions, please select "prefer not to answer."

MH1 Has a trained health professional* ever told you that you have any mental illness?
*A “trained health professional” may be a clinical care provider like a primary care provider, obstetrician, or pediatrician or it may be a mental health specialist, such as a psychiatrist, psychologist, psychiatric nurse practitioner, licensed counselor or social worker

- Yes (1)
- No (2)
- Prefer not to answer (3)

Display This Question:

If Has a trained health professional ever told you that you have any mental illness? *A “trained he... = Yes*

MH2 If yes, did you receive some type of treatment, including therapy or medications?

- Medications (1)
- Therapy (2)
- Both medications + therapy (3)
- Neither (4)
- Prefer not to answer (5)

Display This Question:

If Has a trained health professional ever told you that you have any mental illness? *A “trained he... = Yes*

MH3 If yes, how long ago were you first diagnosed (in years)?

____

MH4 How often have you seen a trained mental health professional for mental health care in the past year?

- ◯ I have not seen a trained mental health professional in the last year (1)
- ◯ Once or twice in the last year (2)
- ◯ Once every 1-3 months (3)
- ◯ Weekly or more than once per week (4)

MH5 Would you say that, in general, your overall mental health is:

- ◯ Excellent (1)
- ◯ Very good (2)
- ◯ Good (3)
- ◯ Fair (4)
- ◯ Poor (5)
- ◯ Don’t know (6)

MH6 Was there any time in the past 12 months when you thought you needed treatment or counseling for mental health issues but did not receive services?

- Yes (1)
- No (2)
- Prefer not to answer (3)

Display This Question:

If Was there any time in the past 12 months when you thought you needed treatment or counseling for... = Yes

MH7 If yes, which of the following reasons, if any, contributed to why you did not receive services? [select multiple]

- I could not afford the cost of services (1)
- I did not know where to go for services (2)
- I believed I could handle the problem without treatment (3)
- I was concerned about being committed to a psychiatric hospital (4)
- I was concerned about having to take medication (5)
- Other [please specify] (6) ____
- Prefer not to answer (7)

End of Block: Mental health history and symptom questions

Start of Block: Pregnancy history

Text4 Now we are going to ask you some questions about pregnancy. If you do not wish to answer certain questions, please select "prefer not to answer."

Pregnancy1 Are you currently pregnant?

○ Yes (1)

○ No (2)

○ Prefer not to answer (3)

Display This Question:

If Are you currently pregnant? = Yes

Pregnancy2 If yes, how far along are you in months:

____

Pregnancy3 Have you ever been pregnant before (not including if you are currently pregnant)?

○ Yes (1)

○ No (2)

Display This Question:

If Have you ever been pregnant before (not including if you are currently pregnant)? = Yes

Pregnancy4 If yes, how many times?

____

Display This Question:

If Have you ever been pregnant before (not including if you are currently pregnant)? = Yes

Pregnancy5 If yes, what is the date your youngest child was born? If the pregnancy did not result in a live child, enter N/A

____

PPD1 Have you ever been told you have postpartum depression?

- Yes (1)
- No (2)
- N/A- was never pregnant (3)
- Prefer not to answer (4)

PPD2 Have you ever been treated for postpartum depression?

- Yes (1)
- No (2)
- N/A- was never pregnant (3)
- Prefer not to answer (4)

End of Block: Pregnancy history
